# Factors Affecting Social Support Status of People Living with HIV/AIDS at Selected Hospitals of North Shewa Zone, Amhara Region, Ethiopia

**DOI:** 10.1155/2021/6695298

**Published:** 2021-04-01

**Authors:** Elyas Admasu Basha, Behailu Tariku Derseh, Abate Dargie Wubetu, Nigus Alemnew Engidaw, Kefyalew Dagne Gizachew

**Affiliations:** ^1^Debre Berhan University, Institute of Medicine and Health Sciences Department of Nursing, P.O. Box 445, Debre Berhan, Ethiopia; ^2^Debre Berhan University, Institute of Medicine and Health Sciences Department of Public Health, P.O. Box 445, Debre Berhan, Ethiopia

## Abstract

**Background:**

Globally, millions of people are affected by human immunodeficiency virus (HIV). Acquired immunodeficiency was linked with psychosocial problems, whereby stigma and discrimination are the most common. Therefore, this study was aimed at assessing the level of the social support problem in people living with human immunodeficiency virus (PLHIV) and factors associated with it at selected hospitals of North Shewa Zone, Amhara Region, Ethiopia.

**Method:**

An institution-based cross-sectional study design was employed. A total of 422 PLHIV were involved in the study. A multidimensional perceived social support scale (MPSSS) for the measurement of social support problems was implemented. Systematic random sampling was used to recruit the study population after selecting study areas by lottery methods. Multivariate logistic regression analyses were performed via SPSS software. The statistical association was declared at a *p* value of less than 0.05 in the final model.

**Result:**

The prevalence of poor social support among study participants was 12.6%. Poor adherence to their antiretrovirus drugs was highly associated with poor social support (AOR = 2.06, 95% CI: 1.36, 3.13). Moreover, psychological distress (AOR = 4.67, 95% CI: 2.02, 10.81) and perceived stigma (AOR = 1.78, 95% CI: 1.18, 2.70) were positively associated.

**Conclusion:**

The burden of poor social support is increasingly affecting the lives of PLHIV. Poor social support in PLHIV is more magnified by poor adherence, having psychological distress, and perceived stigma.

## 1. Introduction

Human Immunodeficiency Virus (HIV) and Acquired Immunodeficiency Syndrome (AIDS) is one of the chronic diseases affecting millions of populations all over the world [1]. As of the end of 2018, there were 37.9 million people living with HIV worldwide, with 20.6 million of them in Africa [1]. In the same document, it was reported that 690,000 PLHIV were in Ethiopia. HIV/AIDS was found to be the main driving force for increased years lived with disability (YLDs) in sub-Saharan Africa in a review of YLDs for 301 acute and chronic diseases and injuries in 188 countries [2].

Various researchers have found high comorbidity of mental and behavioral disorders in people living with HIV/AIDS, with depression being the predominant one [3]. Moreover, the prevalence of depression in PLHIV in Iran was estimated to be more than 70% [4]. A meta-analysis of depression in PLHIV has found a global pooled prevalence of 31% with a prevalence of 24% in Africa [3]. A separate review of depression in people living with HIV in Africa has found a pooled prevalence of 36% [5]. Furthermore, another meta-analysis of the prevalence of depression in people living with HIV in Eastern Africa has found a pooled prevalence of 38% with the highest prevalence in Ethiopia (50%) [6]. Unlike the previous meta-analysis, another more specific meta-analysis of depression in people living with HIV in Ethiopia reported the prevalence of depression in Ethiopia was (37%) [7] and 7.8% of PLHIV were psychologically distressed [8].

It is a well-known fact that social support is a crucial protective factor for depression [9–11]. More importantly, social support is crucial for people living with HIV. According to different studies conducted in different regions of the world, social support in people living with HIV/AIDS has found a significant association with stigma [12, 13], diseases progress [14, 15], adherence [16–19], quality of life [19], substance use [16], and different mental problems [7, 20, 21]. Furthermore, social support is reported to be a factor of healthcare utilization in people living with HIV, which can be considered as the main target of the treatment of HIV [22].

Despite the significance of the problem, there is limited information on the social support system of people living with HIV in Ethiopia. With this gap in mind, this research was aimed at assessing the status of social support in people living with HIV in selected hospitals of North Shewa Zone, Amhara Region, Ethiopia. The findings of this research will have a significant input for researchers, policymakers, and service providers in improving the social support of people living with HIV in Ethiopia.

## 2. Materials and Methods

### 2.1. Study Design, Setting, and Population

A cross-sectional hospital-based study design was employed in three selected public hospitals, North Shewa Zone, Ethiopia. Among 3123 people living with HIV/AIDS who were following their ART, adults aged greater than or equal to 18 years were included in this study. However, study participants who were seriously ill had been excluded.

### 2.2. Sample Size Determination and Sampling Procedure

We determined the sample size for this study using a single population proportion formula at 95% of the confidence interval. Since there was no published data showing the proportion of social support problems in these study area settings and nearby, we used the proportion (P) as 50% and the margin of error (d) of 5%. We also included a 10% nonresponse rate. Therefore, the total sample size becomes 422.

There were three different hospitals selected randomly by a lottery method for our study. The sample size was calculated proportionally from each hospital (Debre Berhan referral hospital = 242, Enat district hospital = 152, and Ataye district hospital = 28). Hence, the sample participants were interviewed by using systematic random sampling after determining the sampling fraction (*k* = 7), and the interview was continued every 7 participants after the first participant was selected by a lottery method from each hospital.

### 2.3. Data Collection Instrument

The questionnaire was first prepared in English, then translated into the national language, Amharic, and then, again translated back to English to check for consistency and rephrasing difficult concepts.

### 2.4. Study Variables

Outcome variable: social support status in people living with HIV/AIDs.

Exposure variables: sociodemographic factors: age, sex, marital status, ethnicity, religion, occupation, educational status, and income; clinical characteristics: WHO HIV/AIDs stages, ART drug adherence, opportunistic infections, duration of knowing their HIV status; and environmental factors: stigma and discrimination and substance use (khat, alcohol, and tobacco).

### 2.5. Measurement of the Study Variables

A structured interviewer-administered self-reported questionnaire (SRQ-20) was used to measure psychological distress, which is validated in Ethiopia [23], and the multidimensional perceived social support scale (MPSSS) for the measurement of social support problems [24] was implemented. If a participant scored greater than or equal to 8 out of 20 of the SRQ-20, then he/she was considered as having psychological distresses. Any mean total score ranging from 1 to 2.9 was considered poor support, and a score of 3 to 5 was considered as good support. Questions for assessing the sociodemographic factors, clinical history, and substance use conditions were designed from different pieces of literature. Factors related to ART drugs adherence and perceived stigma due to the HIV-positive status was assessed by the Morisky Medication-Taking Adherence Scale (MMAS, 4 items) and stigma-related experience scale (5 items) [25], respectively. The MMAS consists of four items with a scoring scheme of “Yes” = 0 and “No” = 1. The items were summed to give a range of scores from 0 to 4. Perceived stigma was measured by the 5 items of stigma experiences of HIV/AIDS patients on a five-point Likert-type scale, which is ‘never,' ‘rarely,' ‘sometimes,' ‘often,' and ‘always.' Items are then recoded into binary variables to reflect the presence or absence of each specific stigma experience in the way that ‘never,' ‘rarely,' and ‘sometimes' are recorded as ‘0' to reflect the absence of stigma and ‘often' and ‘always' are recorded as ‘1' to reflect the presence of stigma. Values are then summed across the five items for a scaled score ranging from 0 to 5. So, the increase in the score of scale shows that respondents are experiencing more stigma, and low score shows that respondents are experiencing lower levels of stigma.

### 2.6. Data Quality Control

We assured data quality by designing appropriate data collection tools. The data collection instrument was pretested on 5% (*n* = 22) of the sample size at the Debre Berhan Health Center to avoid information contamination. Language clarity, appropriateness of data collection tools, and estimating the time required and the necessary amendments were considered based on the pretest. Two days of training was given concerning the data collection tool and the data collection process for both data collectors and supervisors. During the data collection time, close supervision and monitoring were carried out by supervisors and the principal investigator to ensure the quality of the data. Finally, all the collected data were checked by the supervisors and investigators for completeness and consistency during data management, storage, and analysis.

### 2.7. Data Processing, Analysis, and Presentation

Before analysis, the data were cleaned, edited, and coded. Any errors identified at this time were corrected after reviewing the original data using the code numbers. Then, we entered the data using Epi-Data version 3.1 and analyzed using SPSS version 16. Descriptive statistics were used to explain the study participants in relation to study variables. Bivariable and multivariable logistic regression analyses were performed to identify factors associated with poor social support. We considered statistical significance at a *p* value of less than 0.05 in the final model. The strength between the outcome and the exposure variables was presented with adjusted odds ratio and 95% confidence interval of the odds.

## 3. Result

### 3.1. Sociodemographic Characteristics of the Study Participants

Since we were able to convince and teach participants about the objective and the significance of the study, all the contacted 422 participants agreed to participate in the study, yielding a response rate of 100%. The mean age of the participants was 39.15 years (SD ± 10.44). Among the participants, 62.8% (265) were females. Around one participant out of four (42% (179)) of the participants were married. The majority (89.3%) of the participants were Orthodox in religion. Almost all (95.5%) were Amhara by ethnicity. Regarding their occupational status, 30.1% (*n* = 127) of them were government employees. Around two-thirds (71.1%) of them were living alone (Table 1).

### 3.2. Clinical, Medication Adherence, Substance Use, and Perceived Stigma Characteristics of the Participants

Regarding the clinical characteristics of study participants, 86% (*n* = 363) had known their HIV status more than 12 months before the time of data collection. Around half of the respondents (50.9%) were with a cluster of differentiation 4 (CD4) count less than 500 cells/*μ*L. More than three-fourth (78.4%) of the participants were in the World Health Organization (WHO) stage I, and among the participants, 11.1% (*n* = 47) had a history of comorbid tuberculosis. More than quarters (29.6%) of the respondents were currently using alcohol. Most (63.3%) of the participants have had good drug adherence status, and 37% of them have perceived stigma due to their illness (Table 2).

### 3.3. Prevalence of the Social Support Problem among People Living with HIV/AIDs

The prevalence of poor social support among PLWHA in this study was 12.6% (*n* = 53).

### 3.4. Factors Associated with Poor Social Support Status among People Living with HIV/AIDs

Multivariable logistic regression analysis revealed that participants with psychological distress were almost 5 times (AOR = 4.67, 95% CI: 2.02, 10.81) more likely to have poor social support status than participants without psychological distress, whereas participants who were married (AOR = 0.49, 95% CI: 0.32, 0.74) were less likely to have poor social support status compared to the nonmarried participants. In addition, participants who did not have adherence for their ART  drugs (AOR = 2.06, 95% CI: 1.36, 3.13) were more likely to have poor social support status than their counterparts. Finally, people living with HIV/AIDs who had perceived stigma (AOR = 1.78, 95% CI: 1.18, 2.70) were more positively associated with poor social support status (Table 3).

## 4. Discussion

People with different chronic illnesses are frequently at increased risk of developing physical, emotional, and social problems. HIV/AIDS is one of the chronic illnesses predisposing patients to those problems. PLWH should adjust their quality of lives to live longer with their illness. As a result, this study was aimed to assess the level and associated factors of poor social support status of people living with HIV/AIDs at three selected hospitals of North Shewa Zone, Amhara Regional State, Ethiopia.

The prevalence of poor social support status (12.6%) in the current study is lower than in the previous studies conducted in New York state of America [26], and Malaysia [27], whose prevalence was 41%, 51.2%, and 40% respectively. These differences might be due to the differences in study areas, duration, and tools used.

Poor adherence to HAART drugs is positively (AOR = 2.06) associated with poor social support in this current study. This factor was also strongly associated with the social support problem in the previous research conducted in different countries [17, 18, 26, 28]. This association might be due to the fact that patients with HIV prefer to restrict themselves from following their appointment or taking their ART drugs due to fear of stigma and discrimination. As a result, there would be a possibility of rapid viral replication and emergence of different opportunistic infections (OIs), further predisposing them to have perceived stigma. Finally, they tend to have poor social adjustment. Perceived stigma is almost 2 times associated with poor social support status which is in line with the previous studies conducted abroad [12, 27, 29, 30] and Ethiopia [31, 32]. This might be due to the fact that PLWH may perceive other people around them are talking about their HIV status and limit themselves from social interaction. Another factor that is associated with poor social support status in people living with HIV/AIDs on ART was being nonmarried (AOR = 2.06, 95% CI: 1.36, 3.11). This is true that support is mandatory for people on challenging life events, especially for chronic diseases such as HIV/AIDS. As this current study showed married participants were less likely to develop poor social support status than the respondents who were either nonmarried, divorced, or widowed. This was also true in the previous study conducted in Iran [33]. Psychological distress was almost 5 times (AOR = 4.67, 95% CI: 2.02, 10.81) more likely to be associated with poor social support status than their counterparts [7, 20, 21]. Psychological problems secondary to HIV/AIDS further make people living with HIV/AIDs withdraw from different social events. This is true that the characteristic features of psychological problems such as depression are lack of communication/interaction.

## 5. Conclusions

We found a (12.6%) prevalence of poor social support status. Psychological distress, perceived HIV stigma, being nonmarried, and poor adherence were found to be significantly associated with poor social support in this study population.

## Figures and Tables

**Table 1:** Sociodemographic characteristics of study participants at selected hospitals' ART clinics, North Shewa Zone, Amhara Regional State, Ethiopia, 2017 (*n* = 422).

Variables	Frequency, *N* (%)
Age	
18–24 years	22 (5.2)
25–34 years	110 (26.1)
35–44 years	274 (64.9)
≥45 years	16 (3.8)
Sex	
Male	156 (37)
Female	266 (63)
Ethnicity	
Amhara	403 (95.5)
Others	19 (4.5)
Religion	
Orthodox	378 (89.6)
Muslim	17 (4)
Protestant	27 (6.4)
Educational status	
Illiterate	104 (24.6)
Only read and write	46 (10.9)
Primary school	82 (19.4)
Secondary school	99 (23.5)
College diploma and above	91 (21.6)
Marital status	
Married	179 (42.4)
Single	70 (16.6)
Divorced	72 (17.1)
Separated	38 (9)
Widowed	62 (14.7)
Occupation	
Governmental employed	128 (30.3)
Unemployed	63 (14.9)
Daily laborer	26 (6.2)
Housewife	85 (20.1)
Farmer	31 (7.3)
Merchant	61 (14.5)
Others	28 (6.6)
Monthly income	
<300 ETB	107 (25.4)
300–1000 ETB	126 (29.9)
1001–2500 ETB	83 (19.7)
>2500 ETB	106 (25.1)
Living condition	
Living with family	112 (26.5)
Living alone	300 (71.1)
Others	10 (2.4)

**Table 2:** Clinical, medication adherence, and substance use characteristics among PLHIV at selected hospitals' ART clinics, North Shewa Zone, Amhara Regional State, Ethiopia, 2017 (*n* = 422).

Variables	Frequency (*n* = 422) (%)
Last CD4 count	
<500 cells/dl	215 (50.9)
≥500 cells/dl	207 (49.1)
WHO stage	
Stage I	331 (78.4)
Stage II	80 (19)
Stage III	11 (2.6)
Opportunistic infection	
Yes	54 (12.8)
No	368 (87.2)
Types of OIs	
TB	47 (87.04)
Herpes zoster	7 (12.96)
Current tobacco use	
Yes	8 (1.9)
No	414 (98.1)
Current khat use	
Yes	14 (3.3)
No	408 (96.7)
Current alcohol use	
Yes	126 (29.9)
No	296 (70.1)
Drug adherence (ART)	
Yes	269 (63.8)
No	153 (36.2)
Perceived stigma	
Yes	159 (37.7)
No	263 (62.3)

**Table 3:** Factors associated with poor social support among PLHIV at selected hospitals, North Shewa Zone, Amhara Regional State, Ethiopia, 2017 (*n* = 422).

Variables	Poor social support		*p* value	COR (95% CI)	AOR (95% CI)
	Yes	No			
Marital status					
1. Others^*∗∗∗*^	138	104	0.001	2.09 (1.41, 3.09)	2.06 (1.36, 3.11)^*∗∗*^
2. Married	70	110		1	1
Alcohol use					
1. No	15	281		1	
2. Yes	18	208	0.006	1.80 (1.18, 2.75)	—
Adherence level on ART					
1. Poor	93	60	0.01	2.08 (1.39, 3.11)	1.79 (1.15, 2.81)^*∗*^
2. Good	115	154		1	1
Perceived stigma					
1. Yes	92	64	0.033	1.86 (1.25. 2.78)	1.59 (1.04, 2.43)^*∗*^
2. No	116	150		1	1
Psychological distress					
1. Yes	17	28	0.000	7.69 (3.59, 16.50)	4.67 (2.019, 10.805)^*∗∗*^
2. No	36	341		1	1
CD4 count					
1. <500	115	95	0.026	0.65 (0.44, 0.95)	—
2. ≥500	93	119		1	

Significant association: ^*∗*^, *p* value <0.05 and ^*∗∗*^, *p* value<0.01. Others: ^*∗∗∗*^single, divorced, separated, and widowed.

## Data Availability

The data used to support the findings of this study are included in this manuscript.

## Abbreviations

AIDS: Acquired immunodeficiency syndrome
AOR: Adjusted odds ratio
ART: Antiretroviral therapy
HAART: Highly active antiretroviral therapy
HADS: Hospital anxiety depression scale
HIV: Human immunodeficiency virus
OIs: Opportunistic infections
PLWH: People living with human immunodeficiency virus
SRQ-20: Self-report questionnaire-20
WHO: World Health Organization.
